# 

**Published:** 1970-10

**Authors:** 


Bristol Medico-Chirurgical Journal Vol. 85 (iv) No. 316

### CONTENTS

Some Impressions of a New Medical School in Africa ... ... ... 89

J. M. Naish

On the History and Growth of the Bristol Medical School Library ... 93
A. E. S. Roberts

The Problems of Anticoagulant Control — 1. Drug Interactions ... ... 101
T. J. Hamblin

This Week in Parliament ... ... ... ... ... ... ... 105

John Clutton-Brook

Obituary ... ... ... ... ... ... ... ... ... 106

Wisdom from the Past ... ... ... ... ... ... ... 108

News and Notes ... ... ... ... ... ... ... ... 110

F. Bailey & Son Ltd., Dursley, Glos.

